# Effect of WHO-SCC based intra-department mentoring program on quality of intrapartum care in public sector secondary hospitals in Andhra Pradesh, India: Pre-post mixed methods evaluation

**DOI:** 10.1371/journal.pgph.0000530

**Published:** 2022-08-16

**Authors:** Samiksha Singh, Nanda Kishore Kannuri, Aparajita Mishra, Leena Gaikwad, Rajan Shukla, Mukta Tyagi, Swecha Chamarthy

**Affiliations:** 1 Indian Institute of Public Health-Delhi, Public Health Foundation of India, Delhi, India; 2 Indian Institute of Public Health-Hyderabad, Public Health Foundation of India, Hyderabad, India; Sheffield Hallam University, UNITED KINGDOM

## Abstract

Quality of intrapartum care is essential for improving pregnancy outcomes; several models for improving performance are tested, globally. *Dakshata* is one such WHO SCC-based national program—improving resources, providers’ competence, and accountability—in public sector secondary care hospitals of India. Andhra Pradesh state devised strategy of mentoring by the handpicked member from within the obstetric team, supported by external technical partner. We evaluated the effectiveness and assessed contextual factors to success of the program. We conducted pre and post mentoring mixed-method surveys to evaluate the change in evidence-based intrapartum and newborn care practices and stillbirth rates, across 23 of 38 eligible hospitals. We directly observed obstetric assessments and childbirth, extracted data from casesheets and registers, interviewed beneficiaries and conducted facility surveys. We in-depth interviewed stakeholders from state, district and facility managers, mentors and obstetric staff, and external managers for theory-driven qualitative assessment. After one year we found, average adherence to practices sustained high during admission (81%, 81%); improved during childbirth (78%, 86%; p = 0.016); moderate within one hour of birth (72%, 71%), and poor postpartum care before discharge (46% to 43%). Stillbirths reduced from 11(95% CI, 9–13) to 4(3–5) per 1000 births (p<0.001). Some practices did not improve even after sustained reinforcement. Commitment from state, engaging district officers, monitoring and feedback by external managers enabled supportive setting. The structured training and mentoring package, and periodic assessments delivered under supervision ensured the standards of mentoring. The mentoring model is acceptable, effective, less costly and scalable; appears sustainable if state commits to institutionalising a long-term mentoring with adequate monitoring. We conclude that the SCC-based mentoring and skill building program showed improvement in practices during childbirth while it sustained high levels of care during admission, but no improvement in postpartum care. The state needs to monitor and ensure continuous mentoring with required infrastructural support.

## Introduction

Globally, India contributes to 12% (35,000) of maternal deaths [1] and 21% (522,000) neonatal deaths [2] annually. India showed substantial improvement in maternal health outcomes [3–5], but far from MDG targets in 2015 [6]. Despite several initiatives and more than 80% hospital deliveries, the Maternal Mortality Ratio (MMR) is at 113 per 100,000 [4] and Stillbirth rate at 4 per 1,000 live births [7]. The Government of India now emphasises on strengthening infrastructure and quality of obstetric care through programs such as *Dakshata* (2015) package of mentoring for skills [8]; *Pradhan Mantri Surakshit Matritva Abhiyan* (2016) to improve access to quality antenatal care for high-risk conditions [9]; *Laqshya* (2017) to standardise labour rooms, quality certification and quality improvement cycles [10]; *Daksh* skills labs (2018) for upgrading skills [11]; Safe delivery App provides standardised clinical protocols on phone at hand [12], and pilot professional Midwifery (2019) in states [13].

This research evaluates *Dakshata* program [8]—a nationwide scale-up of WHO Safe Childbirth Checklist (SCC) [14], adopted into a 29-item facility-based training tool—to improve care during child-birth and immediately after. In India, the checklist was first pilot tested in 2012 to assess the acceptability of, and behaviour change due to the WHO SCC in the states of Rajasthan, Karnataka and Uttar Pradesh [15–18]. The Better Birth project in Uttar Pradesh, had built in coaching along with use of SCC, and showed improvement in adherence practices but neither on maternal nor newborn mortality [16, 19, 20]. While the SCC pilot in Rajasthan (2012–15) showed decrease in early neonatal mortality [20], and was thus scaled-up into a national program. *Dakshata* incorporates improving resources and clinical skills along with the use of WHO SCC, in a program mode led by the state [21]. Madhya Pradesh, Rajasthan and Andhra Pradesh were among the very first states to initiate the program with support from a technical partner. In Andhra Pradesh, they identified and empowered in-service mentors for training and mentoring as a scalable model while external mentors were used in the other states. We evaluated the intra-department mentoring intervention in Andhra Pradesh as there was need to assess the effect of this state-run program, its accountability, sustainability and scalability for adoption in other states.

### Intervention- *Dakshata* program in Andhra Pradesh

Financially supported by CIFF, JHPIEGO-India office provided technical support to the central government and priority states to implement *Dakshata* program. The intervention package included; a) bulk training of 3 days for use of SCC for essential practices in the labour room, b) mentoring and support (MSV) package of 3–4 months on-site pulse trainings, c) technical support required to ensure availability of resources, and d) technical support to state and country for strategic planning, and monitoring. State managers from JHPIEGO provided planning, administrative and monitoring support to the state and district health departments, and training to government trainers and mentors using standard package and pedagogy skills. Mentors were from within the obstetric team in the facility who provided mentoring under supervision of JHPIEGO program managers. Based on WHO-SCC [14], *Dakshata’s* skill building focused on practices from four crucial pause points: Pause point 1 at the time of admission; Pause point 2 just before and during childbirth; Pause point 3 immediately after childbirth (within 1 hour) and; Pause point 4 at the time of discharge [14, 21].

The program was implemented simultaneously in all 50 selected public sector hospitals across all 13 districts. These included the Medical colleges (12), District hospitals (7), Maternal and Child hospitals (3), Sub-district hospitals (18), and Community health centres (10). Although the program was designed for the secondary and primary level of care, the state government insisted on including the medical colleges too. The program initiated in late 2016 with training of Government trainers who then trained the obstetric staff from the selected facilities under the supervision of JHPIEGO managers, using the structured package. Bulk trainings completed by June 2017, thereafter was a 6 month programmatic delay and the mentoring could only be initiated till January 2018. By September 2018, all the facilities had received two complete cycles of mentoring package, and continued with only need-based mentoring till the phase out at a later date in August 2019.

As per the program hypothesis, the bulk trainings improved providers’ knowledge and skills. The pulse mentoring within provider’s work environment further improved the skills and facilitated easier adoption of essential practices. We thus assessed the effectiveness of an intra-department mentoring program on skill upgradation and use of SCC in labour rooms of District and Sub-district hospitals.

Table 1 describes the implementation process for training, mentoring and periodic assessments.

**Table 1:** Summary description of intervention in Andhra Pradesh as on January 2019.

Mentoring and Support Model: facility mentors mentored other staff using standard package	
State engagement	Between 2015 and 2017 the program evolved from only WHO-SCC based training project to a State-led *Dakshata* program. The State sanctioned resources for 3-day training readily but was not convinced on recruiting new mentors. With deliberations evolved the strategy of intra-department mentors selected from the existing obstetric teams, with an additional honorarium. This resulted in a gap of about 6 months between training and on-site mentoring.
Intervention hospitals	The state insisted intervention in only 50 public sector hospitals with highest childbirth load; Medical colleges, District hospitals, Sub-district hospitals and Community health centres.
Support from technical partner	The JHPIEGO team led by 1 State Program manager and 3 District Program officers provided direct support to the state and districts and indirect support to the hospitals through intra-department mentors. The JHPIEGO team conducted the first rapid assessments for infrastructure and resources and suggested improvements for better preparedness before trainings. They trained government identified *Dakshata* trainers and deputed in-service mentors, supervised and provided handholding support, and conducted periodic assessments at the facility level.
Training	Training included use of checklist and clinical practice those listed in the program. JHPIEGO team helped in micro-planning and facilitated the operations as per the roster. The *Dakshata* trainers trained eligible staff in all selected hospitals under supervision of JHPIEGO managers, but less than 80% eligible staff could be trained. Later several trained staff transferred between hospitals and departments. Thus the state modified the strategy to repeated onsite mentoring and support for any new recruit/ transfer in the labour rooms.
Selection of the mentors	All the 50 program hospitals had designated in-service hospital-based mentors (doctors/ nurses). The mentors were hand-picked after deliberations between the facility leaders, and the technical partner, based on the staff involvement and performance during the initial 3-day training, leadership and communication skills, self-motivation, and upon consensus of the obstetric team. The mentors were a mix of doctors and nurses. JHPIEGO managers provided orientation in a two-day workshop on mentoring roles and pedagogic skills, and handholding support for the initial months of mentoring.
Mentoring	Mentoring consisted of pulse training, mock-drills, and feedback for corrections in practices. Mentors also consulted to ensure availability of essential resources. They used a 5 visit standard package for mentoring. Once this package was completed the facility level mentors repeated the same package once more. Later they continued to provide need-based mentoring, based on the specific gaps identified from periodic assessments.
Periodic assessments	The assessments included scoring the performance (using a composite score), identifying gaps, providing feedback and implementing corrections. Initially, JHPIEGO managers conducted assessments but later they trained and supervised the hospital mentors. The mentors used mobile-based software application that provided a summary report in real-time which was also available to the program managers at district and state. This, additionally, streamlined the monitoring process from the state to the facility.
Monitoring and supervision by state	Mainly the team from JHPIEGO monitored and provided feedback to the state and district department. There was no active supervision from the state. The district nodal officers for *Dakshata* (also responsible for *Laqshya*) were involved in supervision. The state, nonetheless, acknowledged the better performing team/mentor based on District’s feedback.
Phase out	Phase-out was planned for August 2019. The mentors were from public system and were expected to be self-sufficient. The state and district administration acquired management, supervision, monitoring. We, due to logistic constraint, did not assess post-phase out status.

## Methods

### Study design

**Fig 1:**
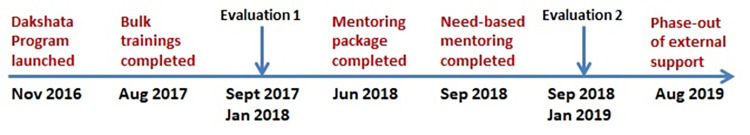
Status of the *Dakshata* program implementation, and evaluation in Andhra Pradesh between 2016 and 2019.

Pre and post mentoring cross-sectional surveys: first assessment from September 2017 to January 2018 and second from September 2018 to January 2019 (Fig 1). We used mixed methods to assess adherence to outcomes, and understanding of the process and implications. We had no role in development or implementation of the intervention. The evaluation was guided by the MRC framework for development and evaluation of complex interventions, and was in accordance with Clinical Trial Rules-2019 issued by the Ministry of Health and Family Welfare, India and, National Ethical Guidelines for Biomedical and Health Research Involving Human Participants (2017) issued by Indian Council of Medical Research.

### Study population

Pregnant women in labour and their newborns who delivered in, and health staff (doctors and nurses) working in the labour rooms of secondary public health hospitals assigned for *Dakshata* program. We also interviewed JHPIEGO program managers, facility, district and state administrators responsible for delivery of *Dakshata* program between 2017 and 2019. The evaluation team and the implementing partner mutually decided to exclude the Medical colleges from this evaluation, as the program was not designed for the medical colleges.

### Sample size and sampling

We estimated the sample size for cross-sectional pre-post assessments for cluster (facility) sampling [22]. We used the percentage adherence to evidence-based practices from baseline of another study from Andhra Pradesh [23]. We computed the sample size for each of the evidence-based practices to detect the desirable change (increase to 80% adherence from baseline) with 80% power and 95% confidence, intra-cluster correlation of 0.1 and cluster size 18. Per facility, we included a higher cluster size (based on feasibility) for less prevalent practices such as—50 case sheets and 30 post-natal mother interviews for obstetric complications and post-natal care; and 400 labour room register entries for stillbirth rates. We got a largest sample size of 22 facilities (out of eligible 38). We conducted stratified sampling to achieve the above stated sample size. We randomly selected 7 of the 13 districts, stratified for region and NMR. We included all the program facilities form these districts, i.e. a total of 23 facilities.

For qualitative assessment, we selected two districts purposely—Vizianagaram is an aspirational district on the northern coast with poorer health indicators, and thus receives higher focus for improvement by the national and state governments, while Kurnool in the south of main land is not an aspirational district [24]. We interviewed sample of all the key stakeholders from state, district and facility managers, mentors and obstetric staff, and external program managers (total 71 and 56 in two assessments).

### Data collection

We reviewed *Dakshata* program’s recommended package to identify 20 key indicators for assessment; 5 indicators per pause point (Table 2 on results), however indicators pertaining to clinical management of the complications could not be analysed due to very small numbers of complications and incomplete or improper documentation. We directly observed practices for Pause points 1 to 3 using checklists and interviewed pregnant women after 24hrs of birth in the post-natal and post-op wards for Pause point 4 using structured interview guide. The direct observations were non-participatory and were conducted throughout days and nights, consecutively including all the eligible clients until the sample size was met, same for post-natal interviews. Post-discharge, we extracted information from casesheets onto a checklist. If the number of discharges were short of 50 during our stay, we included remaining from prior dates going backwards. We extracted information on caesarean, stillbirth and referral rates from childbirth and referral registers in labour rooms or obstetric wards. We also conducted a facility survey to assess for availability of human resource, equipment, drugs and supplies, sanitation and general infrastructure. Qualified nurses who were provided intense training of 5 days collected the data, supervised by team leads. Typically it took 5 days to 15 days to complete data collection per facility. We pilot tested all the quantitative data collection tools before use and developed into an android based application for recording data; and built-in logic checks and restrictions to minimize wrong entries. We used Lenovo tablets for collecting data in the field and uploaded after finishing data collection from each facility. Data were directly saved on the server from where the central research team extracted data on Stata 14.0.

**Table 2:** Cluster level average adherence for each practice, pause points and overall, over time.

	*Bulk trainings completed*	*MSVs completed*	*p value*
Clinical practices observed	Time-1	Time-2	Longitudinal- Linear mixed regression for cluster level
	**N = 462**	**N = 471**	
Blood pressure measured (obs)	90 (84–96)	93 (88–99)	0.310
Foetal heart sounds assessed (obs)	92 (86–97)	97 (95–100)	*0.062
PA examination (obs)	96 (92–99)	98 (96–99)	0.293
PV examination (obs)	95 (91–100)	98 (96–100)	0.238
Hand Hygiene in PV examination (obs)	33 (24–43)	16 (4–28)	0.060
***Pause 1*: *Average of adherence (per facility) to practices***	**81 (77–85)**	**81 (77–84)**	**0.864**
** *Average of adherence excluding hygiene***	**93 (89–97)**	**97 (85–99)**	**0.071**
	**N = 420**	**N = 415**	
Pre-filled oxytocin (obs)	74 (65–83)	91 (85–97)	0.005
Ready bag and mask (obs)	77 (66–88)	94 (89–99)	0.004
Clean, dry and warm towels (obs)	72 (56–87)	59 (45–73)	0.120
Used clean cord cut (obs)	97 (92–100)	99 (98–100)	0.447
Oxytocin within 5 minutes (obs)	71 (63–79)	88 (84–93)	<0.001
***Pause 2*: *Average of adherence (per facility) to practices***	**78 (72–85)**	**86 (82–90)**	**0.016**
Baby dried immediately (obs)	91 (84–98)	99 (97–100)	0.029
Baby weight observed (obs)	96 (93–99)	86 (76–95)	0.037
Breast feeding initiated within one hour (obs)	56 (44–67)	54 (42–66)	0.702
Assessed uterine tone (obs)	67 (55–79)	66 (54–79)	0.959
Mothers vitals checked (obs)	49 (33–64)	48 (34–62)	0.953
***Pause 3*: *Average of adherence (per facility) to practices***	**72 (63–81)**	**71 (64–77)**	**0.723**
	**N = 1,151**	**N = 1,198**	
Newborn immunised (cs)	23 (8–39)	45 (29–61)	0.018
Mother temperature measured (cs)	61 (48–74)	58 (46–71)	0.880
	**N = 1,094**	**N = 1,020**	
Counselled for any danger sign in newborn (int)	40 (26–55)	26 (9–44)	0.304
Counselled for any danger sign in mother (int)	42 (28–57)	32 (16–48)	0.395
Counselled for family planning (int)	62 (52–72)	52 (41–62)	0.169
***Pause 4*: *Average of adherence (per facility) to practices***	**46 (38–54)**	**43 (32–53)**	**0.724**
***Overall average (all 20 practices)***	**69 (64–74)**	**70 (65–75)**	**0.710**
***Overall average excluding pause point 4 (15 practices)***	**77 (72–83)**	**79 (75–83)**	**0.385**

obs = obsevations, cs = casesheets, int = interviews. We calculated average of proportions of adherence in a facility weighted for monthly delivery load per facility. 95% C.I. are mentioned in parenthesis. We conducted linear regression for effect of time on the adherence, weighed for monthly delivery load per facility.

*After adjusting for type of facility, p = 0.041.

We designed In-depth Interview (IDI) guides, on the central idea of efficiency, effectiveness, institutionalization, accountability, sustainability, and scalability. We developed guides for different cadres realising that they have different roles and levels in the intervention. We translated the tools into local language (Hindi and Telugu) and back translated them in English, and pilot tested them. We conducted interviews in providers’ work place/administrators’ offices/wards. The interviews with managers, doctors and nurses were conducted by the trained team of two researchers led by the co-investigator, all of whom were social-anthropologists in health sector. Beneficiary interviews were conducted by two trained research staff with rich research experience. We audio recorded the interviews, unless the consent was denied, and took hand notes. The team transcribed, translated and randomly validated the recordings. Some of the respondents didn’t provide consent to conduct a formal interview so we only had informal discussions and gathered some understanding.

### Ethics

We obtained scientific and ethics approval from the technical and ethics committee from the Indian Institute of Public Health-Hyderabad (IIPHH/TRCIEC/088/2017), and conducted this research in accordance with Declaration of Helsinki and Good Clinical Practice. We obtained informed consent from hospital authorities and service providers for in-facility observations. We obtained written informed consents before interviews. During direct observations, we did not obtain any consent from the pregnant women as the observations did not require us to interact with them in any manner, and we did not want to interfere with routine process of care. The IEC waived informed consent from pregnant women for the observations.

### Analysis

We managed and analysed data on STATA 14.0. For each practice, we computed the proportion of adherence for a facility (cluster) and then computed average of these proportions weighted for monthly delivery load in the facilities. We also computed average adherence to the five practices for each pause point, and overall average adherence for the 20 practices. We conducted linear regression for testing average of proportions over time; poisons regressions for stillbirths and newborn referrals. We also scored performance of facilities, and gave score of one if the adherence to a practice was 80% or more. A facility could get a maximum score of 20 overall, and 5 for a pause point. A score of 14 or more overall, and 4 or more in pause points was considered satisfactory. We conducted qualitative analysis based on pre-defined themes and sub-themes, and also identified new emerging themes. We used Atlas-Ti to code the interviews. During first assessment, we gauged stakeholders’ perspectives about relevance, efficiency, and effectiveness of *Dakshata* and during second assessment we captured information on accountability, sustainability and scalability of the program. We present integrated qualitative results from both times.

## Results

### Facility survey

During first assessment, only half the eligible staff from the labour room was trained under *Dakshata* program. There were also several staff rotations/ transfers but continuous mentoring ensured training of all the untrained/ newly posted obstetric staff. The delivery load in the study facilities marginally increased. At both times, essential trays and equipment were available in almost all labour rooms; newborn thermometer was available only in two-thirds. Availability of essentials for hygiene and clinical management protocols improved except for PPH (S1 Table).

### Adherence to practices

At the time of admission (pause point 1), the adherences to examination practices were already above 90% pre-mentoring, and they stayed high after one year. But the hand hygiene practice prior to vaginal examination halved from 33% to 16% (p = 0.060). The average of all five practices of pause point 1 was the same at 81% both times, and excluding hand hygiene, it was 93% and 97%, p = 0.071. During childbirth (pause point 2), the use of clean cord cut was almost universal. Adherence to pre-filled oxytocin, ready bag and mask, and oxytocin administration within 5 minutes of birth improved significantly. However, the use of clean, dry and warm towels reduced from 72% to 59%. The average adherence for pause point 2 significantly improved from 78% to 86% in one year, p = 0.016. Within one hour of birth (pause point 3), drying the baby immediately after birth improved (91% to 99%) while measuring the baby’s weight reduced (96% to 86%). Early initiation of breastfeeding and mothers’ assessments stayed low. The average adherence in pause point 3 remained similar at 72% and 71%, p = 0.723. The immunisation of newborns before discharge significantly improved from 23% to 45% but was still low. The counselling of the mother for any danger signs and family planning reduced over time (concerns with this evaluation are discussed later). The average adherence for pause point 4 was the lowest and stayed so at 46% and 43%, p = 0.724 in the two assessments. Overall average for all 20 selected practices was same at 69% (95% CI, 64–74) and 70% (95% CI, 65–75), p = 0.710 (Table 2).

Some additional indicators are described in S2 Table. Pre-heating the warmer, keeping the suction device ready before birth, and providing uterine massage after childbirth improved. While monitoring mothers’ blood pressure and foetal heart sounds, delayed cord cut, and newborn and mothers’ temperature after birth decreased.

### Performance scores of facilities

**Fig 2:**
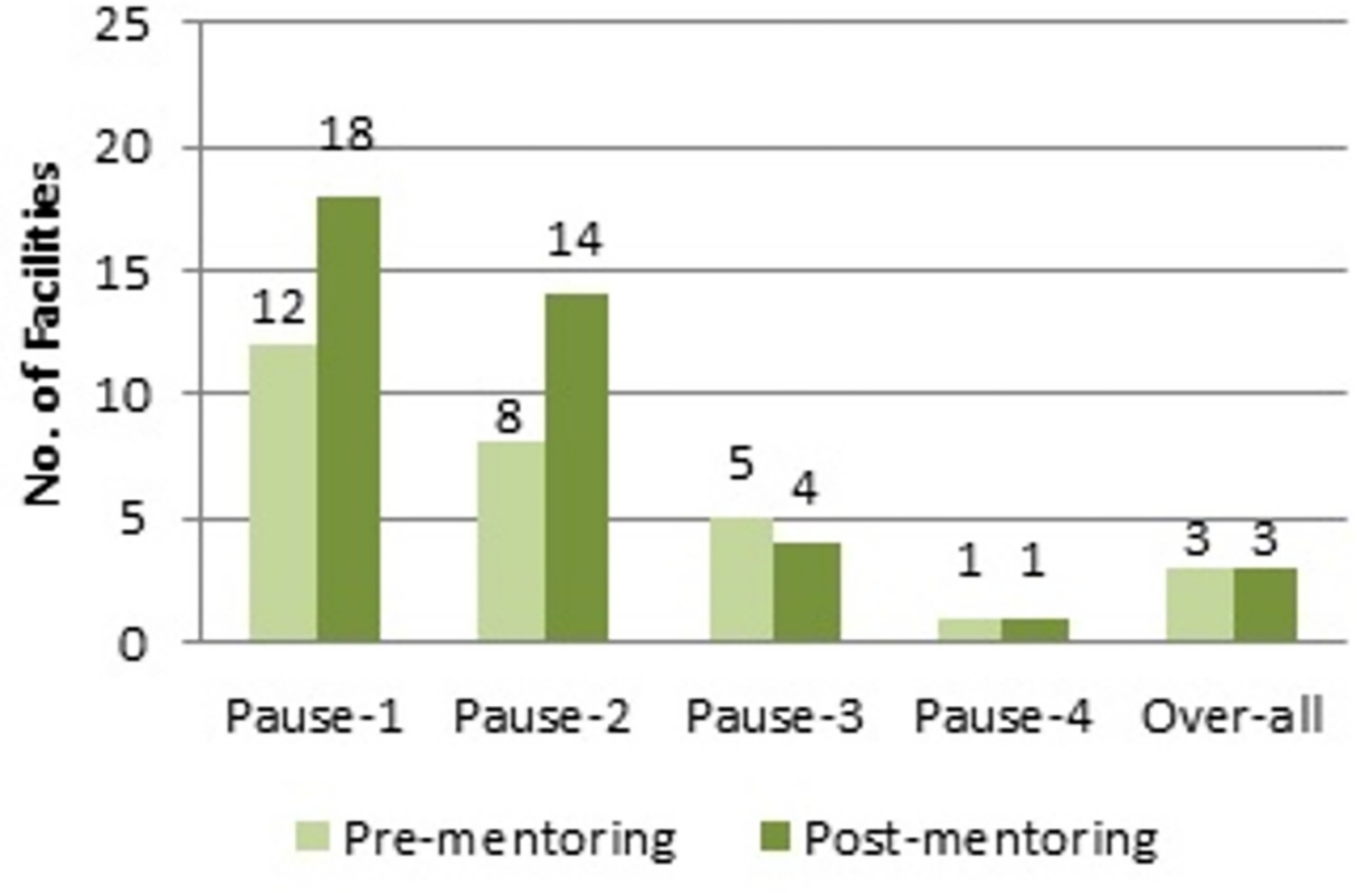
Number of facilities with score 4 or more in pause points and overall 14 or more, over time, N = 22.

We observed that a higher number of facilities scored satisfactory in pause point 1 (12 vs 18) and pause point 2 (8 vs 14) attributable to one year of mentoring and support. Overall only 3 facilities served satisfactory at the two assessments over one year (Fig 2).

### Outcomes

We noted 100 and 41 stillbirths over 3 months at the two assessments. The stillbirth rate reduced over time from 1.1% (95% CI 0.9–1.4) to 0.4% (95% CI, 0.3–0.6) and newborn referral rate from 0.7% (95% CI 1.1–0.4) to 0.1% (0.0–0.1); p <0.001. There were no changes in mothers’ caesarean or referral rates (Table 3).

**Table 3:** Outcomes of obstetric care in the study states, from labour room registers.

	*Bulk trainings completed*	*MSVs completed*	*p value*
	Time-1 N = 9,381	Time-2 N = 10,489	Poisson regression
**Caesarean rate, % (95% C.I.)**	36.6 (35.6–37.6)	35.5 (34.6–36.4)	0.499
**Mothers referral rate, % (95% C.I.)**	0.1 (0.1–0.2)	0.1 (0.0–0.1)	0.401
**Stillbirth rate, % (95% C.I.)**	1.1 (0.9–1.3)	0.4 (0.3–0.5)	<0.001
**Newborn referral rate, % (95% C.I.)**	0.7 (0.6–0.9)	0.1 (0.0–0.1)	<0.001

### Qualitative results

We present these in four broad sections: 1) Effectiveness; 2) Program implementation and State’s ownership; 3) Intensity of support by technical partner; 4) Sustainability and scalability.

#### Effectiveness

All stakeholders believed that the program was very relevant for upgrading skills and improving the quality of care during childbirth. Since the 3-day training, the nurses noted improvement in their knowledge and skills which, they mentioned, continued to improve through-out the mentoring they received in their workplace. The decision-making capabilities and complication management improved. They believed that improved quality of services resulted in reduction in referrals, stillbirths, maternal and newborn mortality. The district and facility administrators acknowledged that *Dakshata* program was the game-changer for the improvement in their respective facilities.

*Resource availability*. Staff noted considerable improvement in infrastructure and availability of resources including clinical protocols that motivated them and facilitated appropriate service delivery. The periodic assessments by the mentors helped them to identify the gaps and rectify the availability as well as the placement of equipment and drugs.

*Quality of care*. Stakeholders stated that quality of services improved, where the quality was expressed in terms of infection prevention (personal hygiene and sanitation, sterilization, biomedical waste management, use of protective gear) and following protocols while assisting vaginal delivery. The service providers stated that the most significant knowledge gained from the program was the complication management (such as postpartum haemorrhage and newborn resuscitation) and early referrals with appropriate pre-referral treatments. The staff also reduced harmful practices such as unnecessary augmentation of labour, fundus pressure, and repeated vaginal examinations, which we also observed. On the other hand, certain practices that staff mentioned had improved were observed to not improve or reduce on direct observation, such as hand hygiene, postnatal check-ups, early initiation of breastfeeding, counselling for exclusive breast-feeding and postpartum care.

The facility and district administrators also noted tremendous improvement in knowledge, practices, and confidence of the staff particularly in the delivery of services during and immediately after childbirth. They also mentioned that the unavailability of staff for post-natal wards was one of the limitations in providing appropriate post-natal care.

*Recording information*. Service providers admitted that prior to *Dakshata*, they did not place much emphasis on recording clinical history, or information from client reports; even diagnosis of complications was recorded for very few. They mentioned that the ongoing mentoring and periodic checks on missing information made them document better; as they also realized it helped in risk identification, early diagnosis, and ease in sharing information between shifts. They, however, were unable to follow complete documentation due to inadequate staff and high workload. The checklist was often entered after admission and after childbirth instead of simultaneously doing so.

*Accountability of service providers*. Stakeholders’ perceived accountability in terms of involvement in, and ownership of, the program components. All stakeholders believed that mentoring and periodic assessments were the main reasons behind service providers being aware and proactive towards their duties, with an increased sense of responsibility. But none of the service providers mentioned using their data efficiently to monitor performance to set accountability.

We noted a positive change in service providers’ attitudes towards providing quality services with a clearer understanding of the benefits. They also mentioned the importance of leadership, external motivation, and encouragement, as well as self-motivation as enablers.

*Work satisfaction*. Nurses were empowered and mentioned an increase in their work satisfaction due to improved competencies. District-level administrators identified concerns with the work satisfaction of the obstetricians and doctors within the existing intricate health system. This did affect the overall team performance.

*Others*. Staff mentioned that there was an inappropriate risk management during the antenatal period in peripheral facilities, thus several patients landed with untreated long-standing complications of pregnancy such as eclampsia, pre-eclampsia, diabetes. Such complications were often not managed at the secondary level and thus led to referral or poor outcomes.

#### Ownership by state health system and program implementation

As this was the program primarily run by the state with support from the external agency, the state’s ownership and engagement was a key factor for implementation and sustainability and scalability.

*Ownership and engagement by state administration*. State health administration sanctioned resources for 3-day training readily but delayed the mentorship. Monitoring was mainly done by the technical partner. We observed no active monitoring by the state for the *Dakshata* quality improvement program. On the other hand, state led and directly monitored implementation of *Laqshya* certification (accreditation) and encouraged the quality of infrastructure and services in labour rooms. Other component of Laqshya, quality improvement cycles, was not yet implemented. District administrators were not satisfied with infrastructure alone and complained about improper allotments of specialists, inadequate technical support, no monitoring and review, and no encouragement and moral support.

*Leadership*. Service providers stated that the significant changes in resources and services were possible due to the active involvement of the district and facility leadership. The leadership, however, stated that this program alone couldn’t bring huge improvements thus they need to also focus on nutrition and adequate antenatal care.

*Efficiency of mentoring and periodic assessments*. The mentors and the program managers stated that the success of the mentoring lay in the standard training of mentors on content and pedagogy, mentoring package, periodic assessment application, and feasibility of frequent follow-ups (mentor being from within the facility). As per the district administrators and technical partner, the identified mentors were clinically capable, had a good rapport and they effectively engaged with the facility leadership and motivated obstetric teams. The program managers mentioned that intra-department mentoring strengthened in-service training as well as reduced the delays in capacity building for new recruits/transfers. The quarterly periodic assessments were considered an essential pillar and integral to mentoring. Although there were some challenges with the resistant and reluctant staff (mainly doctors), and obstetric teams were not yet functional as a quality team.

*Accountability of mentors*. The handholding support from the technical partner was immensely useful and regular follow-up by them improved accountability of the mentors. The JHPIEGO mangers told that the periodic assessment application could be used for monitoring and assigning accountability provided the state and district officers used it too and provided feedback.

*Motivation*, *encouragement*, *awards*. Stakeholders at all levels identified the requirement for establishing mechanisms for motivation and encouragement to service providers to improve services, sustain the achievements, and encourage better performance. They also admitted that motivation need not be in terms of monetary rewards, instead presenting a memento/garland/certificate of appreciation during public meetings could bring marvels in their practices. Self-motivation, on the other hand, differed from person to person and was told to be stimulated with an understanding of the importance of *Dakshata* programme, perceived need for improvement and acknowledgment by seniors. We noted that self-motivation strongly contributed to better performance and accountability at all levels.

#### Intensity of support by technical partner

JHPIEGO state team was the connection between the state, districts and the facilities. The JHPIEGO state team had responsibility for advocacy and persuasion for program strategy, budget and resource allocations by the state.

*Administrative planning and support*. The mid-level administrative support was crucial. They supported all the administrative and managerial planning, conducted supervision and follow-up of the program activities at the state and district. They closely worked with each facility for better preparedness in the labour room and availability of resources via bridging communication between hierarchies, managing supply chain and following up with administrative processes. By the end of one year, the JHPIEGO team supported district administration to take over majority of their tasks.

*Capabilities of JHPIEGO’s team*. All team members were doctors with experience in public health program management. The staff and administrators mentioned that the team from JHPIEGO was highly competent, professional, and motivated. They appreciated the JHPIEGO managers for their expertise and effective way of training and supervision, communication skills, rapport building, problem-solving and innovative approaches to improve quality. The team was noted to be perseverant and approachable. The facility and district leaderships also appreciated the JHPIEGO team’s consistency which was the key to drive their attention and contribution to project activities.

#### Sustainability

We tried to understand the perception of stakeholders regarding the sustainability and scalability of the *Dakshata* program in terms of practices being part of the routine, and the continuation of program components within similar new programs. Service providers described factors that supported the sustainability of the program and provided suggestions (Table 4). A detailed list of facilitators, enablers, suggestions and recommendations for each of the program component are presented in S3 Table.

**Table 4:** Key enablers and suggestions for sustainability of *Dakshata* program as per the stakeholders.

Enablers for sustainability	Suggestions for sustainability
Infrastructure and supply-demand chain of essential resources created an enabling environment and motivation to practice the skills learned.	Sustain and update supplies and resources. Extend to, and strengthen peripheral care facilities under Dakshata program.
New competencies and skills learnt, increased the confidence of the staff. Likely to sustain the practice	Timely trainings and skill up-gradation for the new staff; refresher training for existing staff to reinforce protocols and new evidence.
Improvement in outcomes inspires to sustain or further improve performance. Feedback useful.	Engage and empower the community for better understanding and cooperation to care providers.
Mentoring and periodic assessments at the workplace most essential for continuous quality improvement.	Build systems for mentoring, periodic assessment. Continue periodic visits by an external expert, this will support and ensure the motivation of mentors. The later will reduce the burden on already overburdened administrators.
Supportive leadership and administration, and collective accountability at all the levels of service. Encouragement and motivation.	Structure mechanism for feedback, encouragement, and assigning accountability. Promote a culture of quality improvement.
Monitoring by higher officials and managers essential for program success. Support in problem-solving and addressing resistant uncooperative staff or incoherent quality teams.	At least quarterly monitoring visits and strict follow up. Include Dakshata program review under District and State MCH review meetings, link with outcomes.
Concurrent programs and initiatives that promote quality of care of the pregnant women and newborns shifted focus on the quality of services. Laqshya, and other initiatives contributed to the sustenance of the program by provisions of infrastructure, resources, and skills labs.	The state should lead the program by itself with some support of quality experts. The state should address the gaps in human resources recruitment and allotment. Devise protocols for rationale client distribution, referral system.

The stakeholders unanimously felt the program shall be scaled-up to other districts and states.

## Discussion

This is a robust evaluation of a national program instituting the WHO SCC into a large public health system; reporting results from one of the better resourced and better performing states of India. The stillbirth rates and newborn referral rates reduced attributable to high level of adherence to evidence-based practices during admission and childbirth in this program. Global evidence on use of WHO SCC shows mixed impact on maternal or perinatal mortality [16, 19, 20, 25, 26].

Most published evidence on use of WHO SCC reports improvements in evidence-based practices in initial months of training, particularly when combined with technical trainings, and coaching [26, 27]. But in long term assessments, such as Better Birth trial the practices, that initially improved, reduced when coaching/mentoring was withdrawn [17, 19]. In our evaluated program, the mentoring and other operational support continued for one year and thus the practices in pause points 1,2 and 3 either improved or where sustained at high levels of adherence. For pause point 4, evidence shows improvement but the absolute levels remain low [15, 19], as also found in our evaluation. In this regard, the checklist may require changes considering the challenges such as less human resource and poor felt need for in-facility postnatal care, and poor emphasis on post caesarean care in quality improvement programs in regions such as ours with high caesarean rates. The reduction in stillbirth and newborn referral rates to almost one-third in high load secondary level public health facilities is promising for reducing states PNMR (20 per 1000 live births in 2017 to 14 in 2019 [7]) if *Dakshata* program is continued along with other quality improvement interventions.

The programs supported by state and facility leadership, adequate resources, clinical skills training, coaching/mentoring, and local problem solving are likely to show significant reduction in stillbirths and/or early neonatal mortality [25, 27–29]. We observed an additional drive due to the national program stature, monitoring and supervision by the external agency; standard training of mentors for checklist and clinical skills; structured mentoring package; periodic assessment tools and software application; mentoring by handpicked, self-motivated, capable and acceptable intra-department mentors; recognition and honorarium for mentoring; and the felt need of the nursing staff to learn and improve skills for pause points 1, 2 and 3. We note that after phase out of the partner agency, if the state doesn’t prioritise and actively monitor the program, the district administration will not be committed or in capacity to supervise and sustain the mentoring structure. The state will also need to take decisions on long standing challenges such as frequent rotation of trained staff and administrators, doctor and nurse to patient ratio, and supply chain of resources [27–29]. Further, the persistent change in facility-level practices, patient-centric care, and local problem solving will require long-term commitment and investment into establishing the on-site mentoring and skill building system, incentives and academic paths for mentors, resources in labour rooms, and feedback between administration and care providers. See S3 Table for detailed list of facilitators and challenges to program implementation, local solutions by participants, and deduced recommendations by evaluators (brainstormed with the state and supporting partner).

Rajasthan state implemented *Dakshata* program at the same time and its evaluation also found improvement in pause points 1,2,and 3. Rajasthan is a poorer resourced, and poorer performing state compared to Andhra Pradesh but managed to improve essential resources and practices upto their over 18 months. Similar factors determined effectiveness of the program, but the higher rigour of some contributed to improvement: very strong engagement and monitoring from the state officials with adequate feedback and implementation support by technical partner; prompt lositic support to improve resources and local problem solving; dedicated JHPIEGO mentors; and need-based mentoring at later stages. We concluded that for scale-up, poor resource states like Rajasthan require extensive support, while better performing states such as Andhra Pradesh can be supported by administrative and supervisory support at state, and strong monitoring.

We did not have an opportunity to test the immediate change in practices after initial training; global evidence has established an immediate positive effect in adherence after training [26]. Our evaluation adds evidence on the post-training sustenance and/or improvement in the practices with use of innovative mentoring and monitoring approach. We perceived the Hawthorne effect [30] especially during pause point 1 and in the improved documentation. We did not include measuring temperature and filling up of the checklist because the instructions and resources were haphazard in initial stages thus not comparable with the endline. The timing of measurements for pause point 4 was modified to balance between the actual practice, and the instructions. Several women discharged/left prior to 48 hours, and also due to lack of staff in the postnatal wards, the nurses counselled the new mothers before shifting to postnatal ward or whenever they found chance. We interviewed mothers within 24 hours of childbirth to reduce recall (in both evaluations) which may lead to some underestimation by not including a small fraction that were counselled only at the time of discharge.

## Conclusion

The state appeared ready for a WHO SCC-based skill development and mentoring intervention in public sector secondary hospitals; facilitated and witnessed successful implementation and effect on quality of care at admission and childbirth but no improvement in postpartum care. The stillbirth and newborn referral rates reduced. For sustenance, the state needs to take over monitoring and supervision, ensure adequate skilled human resource, and commit to instituting a long term mentoring mechanism.

## Supporting information

S1 Table Human resource, protocols, and hygiene equipment in the health facilities under study. (DOCX)

S2 Table Adherence to additional practices in Andhra Pradesh, % (95% C.I). (DOCX)

S3 Table Qualitative findings and evaluation recommendations for sustainability and scalability of the Dakshata program components. (DOCX)
