# Supplementary material for: Effect of WHO-SCC based intra-department mentoring program on quality of intrapartum care in public sector secondary hospitals in Andhra Pradesh, India: Pre-post mixed methods evaluation
Source: PLOS Glob Public Health. 2022 Aug 16;2(8):e0000530. doi: 10.1371/journal.pgph.0000530 (PMC10022249; doi:10.1371/journal.pgph.0000530)
Supplement: S1 Table — (DOCX) [file pgph.0000530.s001.docx]

S1 Table: Human resource, protocols, and hygiene equipment in the health facilities under study

| **Attribute** | ***Bulk trainings completed*** | ***MSVs completed*** |
| --- | --- | --- |
|  | **Time 1, N=23** | **Time 2, N=22** |
| Type of facility |  |  |
| - District hospital/ MCH hospital | 7 | 7 |
| - Sub-district hospital | 10 | 10 |
| - CHC/PHC | 6 | 5 |
| Deliveries per month; Mean, Median (IQR) |  |  |
| - District hospital/ MCH hospital | 420,413 (323-490) | 456,423 (382-534) |
| - Sub-district hospital | 242,214 (171-333) | 260,258 (185-334) |
| - CHCs | 126,124 (68-154) | 165,154 (126-194) |
| Human Resource in labour room, Median (IQR) | 3 (1-4) | 3 (1-4) |
| Number of obstetricians, Median (IQR) | 2 (1-3) | 2 (1-3) |
| - Trained in *Dakshata* | 1 (1-2) | 2 (1-3) |
| Number of nurses, Median (IQR) | 4 (3-4) | 4 (3-5) |
| - Trained in *Dakshata* | 2 (1-4) | 4 (3-5) |
| All day functional Caesarean Operation theatre | 87 | 91 |
| Availability of protocols*, % |  |  |
| - Skill Birth Attendance | 43 | 91 |
| - Using Partograph | 83 | 86 |
| - Active Management of 3^rd^ Stage of Labour | 83 | 95 |
| - Using ante-natal corticosteroids | 43 | 82 |
| - Preterm labour | 43 | 86 |
| - PPH Management | 35 | 27 |
| Availability of essentials for infection control, % |  |  |
| - Water in labour room | 96 | 100 |
| - Attached hand-washing area, elbow operated taps | 74 | 95 |
| - Hand washing supplies** | 87 | 91 |
| - Sanitary napkins | 70 | 91 |
| - Bucket under labour table | 87 | 100 |
| - Colour coded bags to dispose biomedical waste | 91 | 95 |
| - Puncture proof container/disposal container | 96 | 95 |
| - Functional Autoclave | 100 | 95 |
| Availability of essential trays, % |  |  |
| - Sterilised delivery tray | 96 | 91 |
| - Sterilised episiotomy tray | 96 | 91 |
| - Newborn tray | 91 | 91 |
| - Emergency drug tray | 91 | 91 |
| - Newborn thermometer | 61 | 68 |
| - Newborn weighing scale | 100 | 100 |
| - Stethoscope | 87 | 95 |
| - Blood pressure apparatus | 91 | 91 |

*Observed displayed protocols, if not available, then enquired about availability of any printed protocols/clinical guidelines kept elsewhere; **Soap, clean towels, alcohol based hand rub. Green highlights for positive change.
