# Supplementary material for: Effect of WHO-SCC based intra-department mentoring program on quality of intrapartum care in public sector secondary hospitals in Andhra Pradesh, India: Pre-post mixed methods evaluation
Source: PLOS Glob Public Health. 2022 Aug 16;2(8):e0000530. doi: 10.1371/journal.pgph.0000530 (PMC10022249; doi:10.1371/journal.pgph.0000530)
Supplement: S2 Table — (DOCX) [file pgph.0000530.s002.docx]

S2 Table: Adherence to additional practices in Andhra Pradesh, % (95% C.I)

|  | ***Bulk trainings completed*** | | ***MSVs completed*** | ***p value*** |
| --- | --- | --- | --- | --- |
| **Pause point-1** | **N=462** | | **N=471** |  |
| All common high-risk assessed (obs) | 34 (18-50) | | 26 (12-39) | 0.418 |
| Any high-risk recorded in the case sheet (obs) | 4 (2-6) | | 4 (1-7) | 0.921 |
| Examined only by nurse at the time of admission | 29 (17-41) | | 34 (19-48) | 0.616 |
|  | **N=1,151** | | **N=1,198** |  |
| Anaemia/ Hb level mentioned (cs) | 95 (94-96) | | 96 (95-97) | 0.178 |
| Progress of labour monitored (cs) | 36 (33-39) | | 29 (26-32) | 0.001 |
| Use of partograph (any entry) (cs) | 31 (28-34) | | 34 (31-37) | 0.229 |
| Any high-risk recorded in the case sheet (cs) | | 13 (10-15) | 11 (9-13) | 0.363 |
| **Pause point-2** | | **N=420** | **N=415** |  |
| Induction of labour using Oxytocin (obs) | | 21 (17-26) | 24 (19-29) | 0.432 |
| Birth companion counselled (obs) | | 70 (61-78) | 38 (25-51) | <0.001 |
| Hand wash before delivery (obs) | | 60 (49-72) | 40 (25-56) | 0.041 |
| Pre-heated warmer (obs) | | 48 (31-61) | 58 (43-73) | 0.315 |
| Ready suction device (obs) | | 92 (84-100) | 98 (96-100) | 0.140 |
| Blood pressure of mother measured (obs) | | 67 (58-77) | 40 (24-56) | 0.005 |
| Foetal heart sounds assessed (obs) | | 69 (62-75) | 44 (32-56) | 0.001 |
| Use of gloves (obs) | | 100 (99-100) | 100 (99-100) | 0.841 |
| Newborn kept on mothers abdomen immediately after birth (obs) | | 80 (75-84) | 84 (80-88) | 0.134 |
| Delayed cord cut (obs) | | 43 (32-53) | 21 (15-26) | <0.001 |
| Uterine massage after childbirth (obs) | | 74 (69-79) | 87 (83-90) | <0.001 |
| Delivered only by nurse | | 76 (71-80) | 76 (61-91) | 0.992 |
| **Pause point-3** | |  |  |  |
| Newborn temperature measured (obs) | | 61 (50-73) | 37 (24-50) | 0.006 |
| **Client centric respectful care** | |  |  |  |
| Mothers informed about Progression of labour | | 85 (83-88) | 70 (67-73) | <0.001 |
| Birth companion allowed during childbirth* | | 87 (84-89) | 91 (88-93) | 0.029 |
| Mother’s privacy maintained during childbirth* | | 95 (94-97) | 88 (85-91) | <0.001 |
| Left unattended by staff for long period (perception of client)* | | 14 (12-18) | 7 (5-10) | <0.001 |
| Good response to any doubts asked by the mother | | 93 (91-94) | 92 (90-93) | 0.281 |
| Overall staff behaviour | |  |  |  |
| - Good | | 93 (92-95) | 88 (86-90) | <0.001 |
| - average | | 6 (4-7) | 12 (10-14) | - |
| - bad | | 1 (1-2) | 0 (0-1) | - |

*Vaginal births only
