# Supplementary material for: Effect of WHO-SCC based intra-department mentoring program on quality of intrapartum care in public sector secondary hospitals in Andhra Pradesh, India: Pre-post mixed methods evaluation
Source: PLOS Glob Public Health. 2022 Aug 16;2(8):e0000530. doi: 10.1371/journal.pgph.0000530 (PMC10022249; doi:10.1371/journal.pgph.0000530)
Supplement: S3 Table — (DOCX) [file pgph.0000530.s003.docx]

S3 Table: Qualitative findings and evaluation recommendations for sustainability and scalability of the *Dakshata* program components

| **Program component** | **Facilitators** | **Challenges** | **Local solutions/ suggestions** | **Evaluation recommendations** |
| --- | --- | --- | --- | --- |
| Service delivery | - Resource availability - Standard protocols - Trainings and repeated mentoring cycles for knowledge and skills upgradation - Assessments and feedback - Nurses empowered by mentoring and an opportunity to participate in problem solving and decision making not restricted due to cadre related hierarchies - In-facility efficient mentor - Self-motivation - Encouragement and awards - Supportive facility leadership - *Laqshya* program for infrastructure upgradation and quality certification | - Inconsistent support from state government - Inadequate budget allocation for resources - Administrative lacunae in implementing corrections - Care immediately after delivery was of less priority - Poor referral mechanisms - Shortage of human resource, overburdened - Rotation of staff, trained staff shifted - Resistant staff; local political interference - Professional (dis-) satisfaction and difficult environment for work - Perceived threat from community as well as from the system, if the case may go worse - Malpractice/ refer to private - Un-supportive labour-room doctor in-charge - Doctor nurse hierarchies, limit QI team functioning - Communities’ knowledge and cooperation in care | - Address staff shortages and resource gaps on priority - In-facility mentoring - Repeated mentoring drives - Restriction on frequent staff rotations - Comprehensive mentoring package, updated timely - Strengthen counselling and patient communication skills - Encouragement and rewards to staff - Formation of quality teams, and peer learning - Strengthen peripheral facilities to shift low-risk delivery load there - Establish collective accountability at all levels - Trainings on problem solving and quality improvement - Long term engagement for establishing quality assurance, control and improvement cycles. | - Sustain resource availability and maintenance, via *Laqshya* quality certification program - Sustain mentoring to sustain improvement in practices, and reinforce change in late modified practices   For long term:   - Establish a culture and norm of mentoring and extend it to newer learnings, as well to other departments - Continuous external support till the culture of quality improvement sets in |
| Mentoring and periodic assessments | - Rigour in selection of mentors - Competency and capabilities of mentors, - Standard content, and pedagogy for trainings and mentoring - In-service mentors, close proximity and continuity - External holding support to mentors - Monitoring of mentors and feedback - Mentors (periodic assessment) IT app handy - Incentives for mentoring - Mentors motivation and accountability - Staff motivation, encouragement and rewards - Facility leader engagement - District level support | - Budget allocation for dedicated mentors - In-service mentors, overburdened - Inter-personal challenges - Resistant staff; political interference - Professional (dis-) satisfaction and (non-) conducive environment of work - Resistant leadership and administration - States role in encouragement, motivation and monitoring the mentors | - Hand-picking the mentors and hand holding support; maintain this process - Appropriate honorarium/ incentives for mentoring - Encouragement and rewards to staff - Comprehensive mentoring package, update timely - Mentors IT application, update and feedback | - Sustainable if political will, long term commitment and integration in existing program - Sustainable only if strong mechanism of monitoring and supervision of mentors by districts and the State - Sustainable in-facility model if there is a mentoring and academic mechanism established in the health system overall; such as exchange programs to medical colleges and provisions of adjunct faculty |
| Data recording, reporting, MIS, review and feedback | - Standard case sheets and registers, including WHO SCC - Encouragement for documentation in case sheets and checklists - Use of mentors app, periodic assessments, feedback - Monitoring and feedback by JHPIEGO program officers | - Infrequent availability of case sheets - Documentation for C-section cases not appropriate - Analysis and use of data not done / absence of skills - No structured mechanism for review and feedback by the State Government | - Embed *Dakshata* periodic assessment and mentors app based information in routine Management Information System (MIS). - Structured plan for review and meetings at the district level | - Sustainable if data monitoring and review mechanism embedded into MIS and district maternal health meetings |
| District level leadership, administration and management | - Government district maternal officers and quality managers support in monitoring of mentoring activities - Necessary infrastructural and resource support made available - Coordinate between relevant maternal and child health programs - Facilitate quality related program activities for *Laqshya* and certifications. | - Too many programs, new protocols too soon, pressurised to perform - Local political interference - Community’s hostile behaviour - Media reporting and propagating failures | - Should at least conduct quarterly monitoring of each facility in jurisdiction - Use mentors app and also review in-facility mentors progress - Integrate *Laqhshya and Dakshata* review meetings and include them mandatorily in monthly District health society meetings - Build district level capacity for management and monitoring | - Sustainable if monitoring led by district leadership and is regularly reviewed in district level meetings.   -Sharing learnings and challenges with facilities in review meetings promotes cross learning, and motivate for better performance   - Capacity building for leadership, admin and monitoring support, to be able to manage the program; hand holding support shall continue |
| State leadership, administration and management | - State willingness and political will to improve quality of obstetric care - Structured guidelines, manuals and tools - Strategic partners support in planning, implementation and monitoring of *Dakshata* program; strong at state and district level | - State administrator not actively engaged in routine management and monitoring - Human resource and infrastructure challanges - State review of *Dakshata* not embedded in routine RCH review meetings | - *Dakshata* program and *Laqshya* to be integrated and reviewed in the State MCH review meetings - Monitor through mentor app and other program indicators | - State leadership and ownership, essential. Need to monitor and review closely. |
