# Supplementary material for: Effect of WHO-SCC based intra-department mentoring program on quality of intrapartum care in public sector secondary hospitals in Andhra Pradesh, India: Pre-post mixed methods evaluation
Source: PLOS Glob Public Health. 2022 Aug 16;2(8):e0000530. doi: 10.1371/journal.pgph.0000530 (PMC10022249; doi:10.1371/journal.pgph.0000530)
Supplement: Attachment — Submitted filename: Response to Academic editor.docx [file pgph.0000530.s004.docx]

**Response to Academic editor**

Journal Requirements:

1. Please provide a detailed Financial Disclosure statement. This is published with the article. It must therefore be completed in full sentences and contain the exact wording you wish to be published. State the initials, alongside each funding source, of each author to receive each grant. Added as advised

This study was funded by the Children’s Investment Fund Foundation. SS, NKS, AM, LG, RS, MT AND SC were supported from this grant. The funder had no role in the study design, data collection, analysis, decision for publication, or preparation of this manuscript.

2. Please update your Competing Interests statement. If you have no competing interests to declare, please state: “The authors have declared that no competing interests exist.” Revised

3. In the online submission form, you indicated that “Most of the data and findings from the evaluation are shared in this manuscript and the supplementary file. The data sets are available from the corresponding author on reasonable request.”. All PLOS journals now require all data underlying the findings described in their manuscript to be freely available to other researchers, either 1. In a public repository, 2. Within the manuscript itself, or 3. Uploaded as supplementary information.
This policy applies to all data except where public deposition would breach compliance with the protocol approved by your research ethics board. If your data cannot be made publicly available for ethical or legal reasons (e.g., public availability would compromise patient privacy), please explain your reasons by return email and your exemption request will be escalated to the editor for approval. Your exemption request will be handled independently and will not hold up the peer review process, but will need to be resolved should your manuscript be accepted for publication. One of the Editorial team will then be in touch if there are any issues.

All the analysed data are made available in manuscript and the supplementary information. The data sets with individual observations and the details of clinical management are not provided as the state health department does not permit publishing them indiscriminately in public.

4. Please provide separate figure files in .tif or .eps format only and ensure that all files are under our size limit of 10MB. For more information about how to convert your figure files please see our guidelines: <https://journals.plos.org/globalpublichealth/s/figures> Edited

5. We have noticed that you have uploaded Supporting Information files, but you have not included a list of legends. Please add a full list of legends for your Supporting Information files after the references list. Added.

6. Please review your reference list to ensure that it is complete and correct. If you have cited papers that have been retracted, please include the rationale for doing so in the manuscript text, or remove these references and replace them with relevant current references. Any changes to the reference list should be mentioned in the rebuttal letter that accompanies your revised manuscript. If you need to cite a retracted article, indicate the article’s retracted status in the References list and also include a citation and full reference for the retraction notice. Checked.

Additional Editor Comments (if provided):

There are minor comments on the body of the paper. You have mentioned in the letter to the Editor that this is a protocol but for me this is pre- and post- analysis of Dakshata program. You are absolutely correct as we have mentioned in the title too. Due to some error I may have mentioned it as protocol in the cover letter. I will correct the cover letter.

Significance level in *,** as well as figures in parentheses are not mentioned in Table 2. Thank you for noticing. We have corrected it.

You have not provided justification for choosing Andhra Pradesh. Justification added at the end of 2^nd^ para in Methods section.

The discussion didn't include comparing results with Rajasthan or any other state where this program got implemented. Thank you for your suggestion. We have added summary results and conclusion from evaluation in Rajasthan, in Discussion section

Finally, provide an estimated future benefits of the programme at the state level and how far this will succeed in reducing stillbirths and NMR. Added

The reduction in still birth and newborn referral rates to almost one-third in high load secondary level public health facilities is promising for reducing sates PNMR (20 per 1000 live births in 2017 to 14 in 2019) with continued Dakshata program along with other quality improvement interventions.

Other points marked on the pdf onsite are also revised. Just mentioning clarification for a few here.

Page 10: later on you stated 20 point scores on p8. The periodic assessment score by mentors was different from evaluation score. I have deleted the ‘19 point’ score mention to avoid confusion.

Page 13: earlier mentioned 19 point scores. Same explanation as above

Thank you for your inputs.

Best regards

Samiksha
